# Investing in health preparedness, response and resilience: a genomics costing tool focused on next generation sequencing

**DOI:** 10.3389/fpubh.2024.1404243

**Published:** 2024-05-09

**Authors:** Oluwatosin Wuraola Akande, Babak Afrough, Maria Amante, Lisa Carter, Jane Cunningham, Noah Clayton Hull, Leena Inamdar, Alexandr Jaguparov, Marco Marklewitz, Biran Musul, Ashley Norberg, Dmitriy I. Pereyaslov, Angela Lee Poates, Gina Samaan, Anita Suresh, Swapna Uplekar, Aude Wilhem, Joanna Salvi Le Garrec Zwetyenga, Toni Whistler

**Affiliations:** ^1^Department of Epidemic and Pandemic Preparedness and Prevention, World Health Organization, Geneva, Switzerland; ^2^New Variant Assessment Platform, UK Health Security Agency, London, United Kingdom; ^3^Infectious Hazard Management, World Health Organization Regional Office for Europe, Copenhagen, Denmark; ^4^Department of Country Readiness Strengthening, World Health Organization Lyon Office, Lyon, France; ^5^Global Health, Association of Public Health Laboratories, Bethesda, MD, United States; ^6^Genomics and Sequencing, FIND, Geneva, Switzerland; ^7^Technical Advice and Partnership Department, The Global Fund to Fight AIDS, Tuberculosis and Malaria, Geneva, Switzerland

**Keywords:** genomic sequencing, cost-analysis, infectious disease, next-generation sequencing, genomic surveillance, costing tool

## Abstract

The world has seen unprecedented gains in the global genomic surveillance capacities for pathogens with pandemic and epidemic potential within the last 4 years. To strengthen and sustain the gains made, WHO is working with countries and partners to implement the Global Genomic Surveillance Strategy for Pathogens with Pandemic and Epidemic Potential 2022–2032. A key technical product developed through these multi-agency collaborative efforts is a genomics costing tool (GCT), as sought by many countries. This tool was developed by five institutions – Association of Public Health Laboratories, FIND, The Global Fund to Fight AIDS, Tuberculosis and Malaria, UK Health Security Agency, and the World Health Organization. These institutions developed the GCT to support financial planning and budgeting for SARS-CoV-2 next-generation sequencing activities, including bioinformatic analysis. The tool costs infrastructure, consumables and reagents, human resources, facility and quality management. It is being used by countries to (1) obtain costs of routine sequencing and bioinformatics activities, (2) optimize available resources, and (3) build an investment case for the scale-up or establishment of sequencing and bioinformatics activities. The tool has been validated and is available in English and Russian at https://www.who.int/publications/i/item/9789240090866. This paper aims to highlight the rationale for developing the tool, describe the process of the collaborative effort in developing the tool, and describe the utility of the tool to countries.

## 1 Introduction

Globally, significant gains have been made in genomic surveillance capacities in response to the severe acute respiratory syndrome coronavirus 2 (SARS-CoV-2) pandemic (1). Between February 2021 and December 2023, the number of countries with next-generation sequencing (NGS) capacity increased from 103 to 167 out of 194. Following the launch of the Global Genomic Surveillance Strategy for Pathogens with Pandemic and Epidemic Potential 2022–2032 (the “Strategy”), World Health Organization (WHO) is driving its implementation in collaboration with countries and partners (2). This Strategy provides a high-level unifying framework to strengthen genomic surveillance capacities to enable quality, timely, and appropriate public health actions within local to global surveillance systems.

Countries are increasingly using NGS technologies to characterize and monitor a wide range of circulating pathogens and developing vaccines, diagnostic tools and therapeutics (3). Work is now required to ensure that countries sustain and strengthen their genomic sequencing capacity. There is a need to expand use cases to leverage existing investments in instruments and operational capacity. Many countries are already embarking on this path (4). However, a significant challenge to sustainable genomics is insufficient financing (4). To achieve sustainable genomics, countries are encouraged to develop a costed national genomic surveillance strategy, which includes financial planning and budgeting (5).

Based on demand from countries and its value-add, one of the technical derivatives of the Strategy is a genomics costing tool (GCT) to support financial planning and budgeting for genomic sequencing laboratories. The tool was jointly developed by the Association of Public Health Laboratories (APHL), FIND, The Global Fund to Fight AIDS, Tuberculosis and Malaria (TGF), UK Health Security Agency (UKHSA), and the WHO. Several institutions had existing, simple costing tools, but the working group acknowledged the need to develop a common approach to support countries in financial planning and budgeting for genomics.

A comprehensive GCT to support short- and long-term financial planning for costing SARS-CoV-2 genomic sequencing infrastructure, reagents and consumables using different instruments, human resources (including training for laboratory sequencing and bioinformatics), facility, and quality management was developed. This tool was launched in December 2023 and is expected to be useful to countries, regional and global policymakers, health administrators and economists, laboratory directors, and quality managers. The GCT is available in English and Russian at https://www.who.int/publications/i/item/9789240090866. This paper aims to highlight the rationale for developing the tool, describe the process of the collaborative effort in developing the tool, and describe the utility of the tool to countries.

## 2 Approach for the development of the genomics costing tool

The GCT was developed over a 16-month period, starting from the establishment of the working group in August 2022 to the official launch of the tool in December 2023.

### 2.1 Establishment of the working group

Recognizing the need to leverage existing assets and partnerships, five agencies with experience in working with countries to strengthen genomic surveillance capacities came together to form a working group. This included experience working on a laboratory-based costing tool, conducting national laboratory needs assessments, market shaping and procurement activities for sequencing reagents and consumables, understanding of laboratory infrastructures needed to build or scale up sequencing and bioinformatics activities, and providing broader country support for genomic surveillance. This group includes representatives with genomics technical and capacity strengthening expertise:

APHL represents state and local governmental health laboratories in the United States; including public health, agricultural, environmental laboratories (6). With over 20 years’ experience in more than 30 countries, APHL builds effective national laboratory systems and expands access to quality diagnostic testing services.

FIND is a WHO Collaborating Centre for Laboratory Strengthening and Diagnostic Technology Evaluation and co-convener of the ACT-Accelerator diagnostics pillar (7). FIND connects countries and communities, funders, decision-makers, healthcare providers and developers to ensure equitable access to reliable diagnosis around the world.

TGF is an international financing and partnership organization that works to fight AIDS, Tuberculosis and Malaria and more recently COVID-19 (8). TGF is also a founding partner of the Access to COVID-19 Tools Accelerator and co-leads its diagnostics pillar.

UKHSA is an executive government agency of the UK’s Department of Health and Social Care (9). Through its New Variant Assessment Platform (NVAP), UKHSA supports global partners to strengthen their genomic sequencing capability and capacity, either by building on existing infrastructure or by conducting sequencing and analysis for variants of concern if requested by a country.

WHO is the United Nations agency that plays an essential role in coordinating global response to health threats and connecting nations, partners and people to improve local health systems (10). Through its headquarters, six regional offices, and more than 150 country offices, WHO fosters country-led initiatives that strengthen genomic surveillance capacities across 194 countries (countries here refer to the 194 WHO Member States) (3). The GCT working group has representatives from the Health Emergencies Programme of WHO headquarters and WHO European Regional Office.

With coordination and leadership from WHO in August 2022, the team developed a strategic workplan to (1) identify and evaluate pre-existing costing tools; (2) determine country costing needs; (3) develop the scope and features of the tool; (4) build the tool and its user manual; and (5) validate the tool through pilot exercises.

### 2.2 Identification and evaluation of pre-existing laboratory costing tools

The working group conducted a landscape review to identify pre-existing tools that could be used to cost sequencing and bioinformatics, or other related laboratory costing tools. Based on this review, four tools were identified and evaluated (Table 1).

**Table 1:** Evaluation of pre-existing laboratory costing tools.

**Costing tools**				
**General information**				
Developer	International governmental agency	National governmental agency	National governmental agency	Non-governmental organization
Public availability	Yes	No	No	No
Genomics specific	No	No	Yes	Yes
Pathogen covered	Agnostic	Agnostic	SARS-CoV-2	SARS-CoV-2
Per sample costing	Per test	Yes	Yes	No
Software used	Microsoft Excel™	Microsoft Excel™	Microsoft Excel™	Microsoft Excel™
**Costed categories**				
Specimen collection and transport	User addition under reagents and consumables	Captures sample packaging and shipping	Captures specimen receipt and extraction without transport	No
Infrastructure and equipment	Captures equipment cost, accounting for amortization	Captures equipment cost, accounting for amortization	Captures equipment costs	Captures equipment cost, including service agreement
Reagents and consumables	Captures user-inputted costs, plus some indicative pricing	Captures user-inputted costs	Provides indicative pricing	Specific for one manufacturer
Bioinformatics	No	No	Yes	No
Workforce	Captures personnel cost, including administrative costs	Captures personnel and benefits cost based on time spent by workflow step	Captures staff salaries and training costs	No
Facility management	Rent and utilities	Captures facility maintenance including utilities	No	No
Quality management	Yes	No	No	No
Others	Accounts for retesting	Accounts for retesting	Nothing additional	Nothing additional
**Output**				
Cost breakdown	Reagents & consumable, equipment, personnel, QMS & facilities per sample	Transport, personnel, supplies, Equipment, and overhead	Personnel and training, reagents and computing supplies, equipment and infrastructure	Equipment and reagent totals by instrument
Final cost	Cost of one test.	Cost per sample & cost per run	Total 1 year cost and cost per sample	Equipment and reagent totals per year
**Ease of use**				
Pre-defined supply list	Not complete, user additions	No	Available	Available
Use of color codes	Yes	Yes	No	Yes
Language	English, French, Russian, Ukrainian	English	English	English

Indirect designation indicates this can be manually added to the tool as a step in the process but is not part of any costing breakdown.

### 2.3 Identification of country costing needs

The working group scoped the “ideal” tool, based on their experience providing country-specific support. Consensus was reached on the need to develop a user-friendly, comprehensive costing tool that allows users to input data specific to their laboratory and produce an output that allows them to identify cost drivers. The outputs are kept simple to allow interpretation by a broader audience, including health policy and budgeting decision makers.

A tool that can adjust for country/laboratory context was deemed critical. It should consider the annual throughput of specimens, sequencing and bioinformatics infrastructure (with instrument amortization and maintenance), reagents and consumables (including shipping and applicable custom clearance fees) with the flexibility of cost adjustments for items, allowing for national variations in pricing.

In addition, the tool should provide cost breakdowns based on categories such as reagents for sequencing and library preparation, consumables (including sample retests), equipment maintenance, workforce, facility, and quality management. Cost breakdown by NGS workflow such as sample receipt, nucleic acid extraction, polymerase chain reaction (PCR) testing, library preparation, sequencing, and bioinformatics was critical. Furthermore, the tool should be able to estimate establishment costs (first year of running a laboratory), and total operational costs (for the following years) – this is particularly useful to institutions aiming to establish their NGS laboratory and help all laboratories identify cost drivers allowing for informed decisions to optimize their workflows overtime.

## 3 Development of the genomics costing tool

The GCT was built on the existing Microsoft Excel™ based Laboratory Test Costing Tool (LTCT) of the Better Labs for Better Health initiative, developed by the WHO Regional Office for Europe (11). The LTCT, released in 2019, supports countries in evaluating and justifying the cost of generic laboratory tests, and assisting in producing pricelists for these tests. It is, however, not tailored to NGS.

The GCT has seven worksheets: data entry and results, reagents and consumables, equipment, personnel and training, facility and transport, bioinformatics, and quality management. The worksheets use cost estimates built on data gathered by the group, or users can input their own data and costs (i.e., price per unit and quantity of reagents and consumables across the workflow steps). For equipment, the tool takes into consideration quantity, unit cost, recommended lifetime years, age of equipment, amortization value, maintenance and calibration costs, and the percentage use for sequencing SARS-CoV-2. The tool offers a range of bioinformatic options aligned with annual sequencing volumes ranging from cloud-based solutions, computer workstations, to high-performance computing servers. This version of the tool is costed in United States dollars. However, users can input their local currency for reference.

The tool allows users to estimate personnel and training costs, based on annual salaries and the percentage of time spent on SARS-CoV-2 sequencing. Similarly, facility services such as rent, electricity, waste management and transportation services (such as sample shipment, insurance, custom clearance) are considered. Quality management activities such as ISO 15189 accreditation, external quality assessment for PCR and NGS, and biosafety cabinet certification can also be costed.

**Figure 1:**
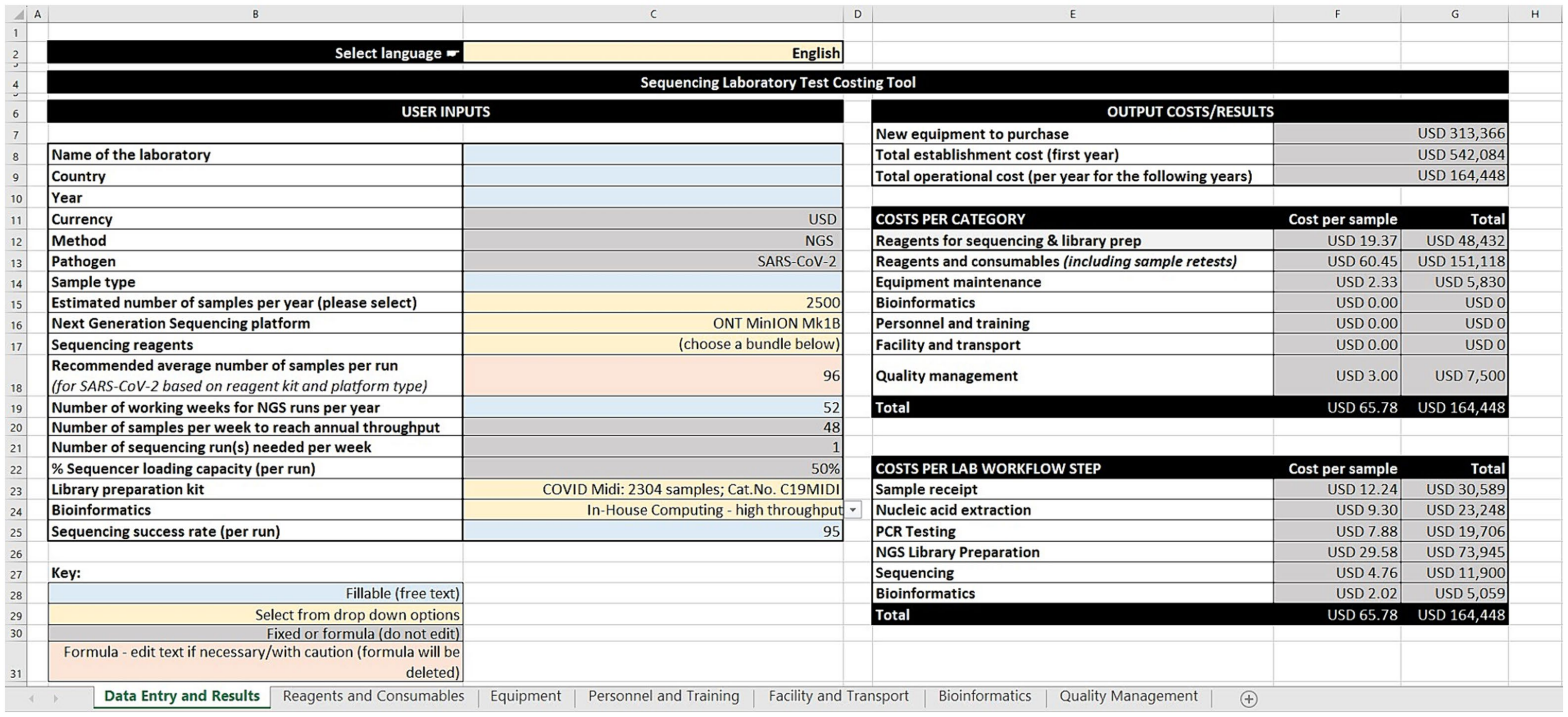
Screenshot of the genomics costing tool data entry and results worksheet.

Following the input across the seven sheets, the output costs/results are generated on the data entry and results worksheet (Figure 1). These are divided into three categories: (1) Cost estimates for new equipment purchases, total establishment cost (first year), and total operational cost (per year for the following years), (2) Cost per category in terms of cost per sample and total cost for sequencing and library preparation reagents, other reagents and consumables (including sample retests), equipment maintenance, bioinformatics, personnel and training, facility and transport, and quality management, and (3) Cost per laboratory workflow step as cost per sample and total cost for sample receipt, nucleic acid extraction, PCR testing, library preparation, sequencing, and bioinformatics.

The draft tool and a manual describing the steps to use it were completed in May 2023.

### 3.1 Validation and finalization of the genomics costing tool and the user manual

Between June and August 2023, the tool was validated through pilot exercises conducted in three laboratories with varying throughput capacities and sequencing and bioinformatics infrastructure in Kyrgyzstan, Ghana and Oman. During the pilot exercises, the following scenarios were tested: (1) validation: to retrospectively obtain the cost of the laboratory’s genomics activities in the previous year; (2) routine costing: to determine the annual cost of routine NGS operational activities in the laboratory; (3) optimization: to help laboratories make informed decisions on how to get better value for money by optimizing their NGS resources; and (4) scale up: to project the cost of scaling up NGS activities from low to high throughput.

Trainers for each pilot were drawn from group members depending on availability and funding. Both on-site and remote participation enabled pilot exercises to be conducted efficiently.

Each participating laboratory filled a pre-pilot survey which collected information to assess the NGS capacity (annual throughput, sequencing and bioinformatics infrastructure, and workforce), pathogens sequenced, and quantity, pricing, logistics and supply chain for reagents, consumables, and equipment. Discussions during the pilot exercises and a post-pilot survey were conducted to collect feedback on the utility of the tool and the user experience.

Feedback and findings from the pilot exercises were used to iterate and improve the tool and the user manual. After the pilot exercises, the tool was pressure tested by a high-throughput European regional laboratory to finalize the tool.

## 4 Preliminary country roll-out and support

As of December 2023, and following its validation in three countries, the tool was rolled out in two countries. Trainings were conducted in Namibia and Georgia in October and November respectively, bringing together a range of technical experts (including laboratory scientists, quality managers, finance, and procurement officers). Participants expressed positive user experiences and acknowledged the utility of the tool in supporting financial planning and budgeting for genomics (12).

## 5 Publication of the genomics costing tool and the user manual

The GCT, alongside its user manual was officially launched on 15 December 2023 during the international meeting on “Sustaining gains in genomics for managing pandemic and epidemic threats” hosted by WHO in Istanbul, Türkiye (13). This hybrid meeting had over 110 country representatives across all the WHO regions, partner agencies and WHO staff. Meeting participants were trained on the use of the tool which is now publicly accessible (14) in both English and Russian versions.

## 6 Discussion

The GCT was developed to support countries in financial planning and budgeting for SARS-CoV-2 genomic surveillance. NGS is a relatively expensive, yet important technology for pandemic and epidemic preparedness, prevention, and response. The GCT supports countries and relevant stakeholders in making decisions that foster sustainable and predictable financing for NGS, particularly within public health systems and considering existing national priorities for laboratory activities.

During the pilot exercises, some impediments to sustainable genomics were revealed. It became evident that sequencing equipment were becoming redundant. A situation attributed by laboratory managers to inadequate financial resources, reduced specimen workflow and limited technical expertise to maintain the equipment. Notably, maintenance and servicing contracts, which are accounted for in the GCT, emerged as significant cost drivers. This demonstrates the need for national and international procuring institutions, including donor agencies to consider the longer-term sustainability of the capacities they support.

The development of the GCT illustrates the power of partnerships toward achieving a common goal. This multinational, interagency collaboration highlights the importance and success of sharing expertise, building trust, and developing networks. These collaborations are critical to pandemic and epidemic preparedness, prevention, and response, and for achieving global health security.

We envisage the utility of the GCT for a wide range of stakeholders from subnational to global levels. Public health laboratories can use the GCT to obtain costs for routine NGS activities, optimize their NGS protocols to get the best value for money, estimate costs for planned scale up, and support an investment case for strengthening and sustaining genomics. The GCT will also be useful to research/academic institutions in supporting accurate budget estimates for grant proposals. The tool will enable appropriate and efficient resource allocation for NGS-related infrastructure, reagents and consumables, and personnel. It allows for comparative analysis, helping laboratories achieve cost-effectiveness across NGS instruments and library preparation methodologies. These analyses would allow the user to make informed choices, optimizing resources.

Furthermore, the tool may be applicable across various sectors, from research to healthcare and beyond, especially once expanded to other pathogens. For example, service providers/clinical laboratories can use the GCT to determine the cost of NGS-based tests, facilitating transparent pricing and affordable healthcare services. Also, biotechnology and pharmaceutical companies could optimize Research & Development budgets, plan drug discovery projects, and evaluate the cost-effectiveness of genomic studies. Venture capitalists and investors can assess the financial viability of NGS-related startups and technologies.

To best serve different players, the tool has been made to be user-friendly, customizable to different workflows, and can be updated to reflect changing costs and technologies, providing cost comparisons across different instruments and methodologies. Additionally, this tool integrates other resources used in NGS, such as data analysis and specimen collection to provide a comprehensive solution for all stakeholders.

### 6.1 Limitations of the tool

The current version (first edition) of the GCT has some limitations. This version is tailored to NGS costing for SARS-CoV-2. The tool’s present iteration can only estimate the cost of a specific sequencing platform and instrument model. This tool is unable to assess laboratories that may have multiple instruments of the same manufacturer and type (e.g., two Illumina iSeq100s). It is also unable to comparatively assess costing for laboratories with mixed sequencing instrumentation (e.g., an Oxford Nanopore Technology GridION and an Illumina MiniSeq). Currently, users could complete this tool multiple times to create the prices for different systems, zeroing out any elements that were captured in the initial iteration (i.e., “Personnel and Training,” “Facility and Transport,” “Bioinformatics” and “Quality Management”). The GCT is limited to Illumina and Oxford Nanopore Technology platforms, which are the most commonly used for SARS-CoV-2 (15). Finally, the tool estimates costs for a range of sample throughput between 600 to 12,000 *per annum*.

### 6.2 Looking ahead

A second edition of the GCT is expected and needed to address current limitations, consider user feedback from the first edition, and be pathogen agnostic. Other broader applications such as wastewater surveillance, drug resistance screening for HIV and *Mycobacterium tuberculosis,* are being considered. The next version will allow for lower sample throughputs, currently at a minimum of 600 samples/annum. In addition, the next version will allow simultaneous costing for multiple sequencing platforms. Sample acquisition and processing costs linked to collection, handling and transport should be available for inclusion as needed, possibly covering sample tracking, barcode labeling, and sample pooling. Lastly, costs associated with implementing and maintaining sample tracking systems and inventory management for reagents and consumables should be included.

## 7 Conclusion

The world has seen significant gains in pathogen genomics capacity since the COVID-19 pandemic and work is now needed to strengthen and sustain these gains. Though there has been a decrease in the cost of sequencing technology in recent years, it remains an expensive technology, particularly in low- and middle-income countries (4). The GCT was developed by five institutions and demonstrates the value of collaboration toward achieving sustainable capacities in genomics. The GCT supports short- and long-term financial planning, budgeting, and resource mobilization for SARS-CoV-2 genomic surveillance for countries, donors, and other stakeholders. This tool will play a key role in supporting sustainable pathogen genomics for the detection, characterization, and monitoring of pathogens, the development of effective countermeasures such as vaccines, therapeutics, diagnostics, and making informed public health decisions. Subsequent versions of the tool will be pathogen agnostic, so that it can be used to cost genomic sequencing more holistically.

## Data availability statement

The original contributions presented in the study are included in the article/supplementary material, further inquiries can be directed to the corresponding author.

## Author contributions

OA: Conceptualization, Data curation, Investigation, Methodology, Project administration, Writing – original draft, Writing – review & editing, Resources, Validation. BA: Conceptualization, Data curation, Investigation, Methodology, Software, Writing – review & editing, Resources, Validation. MA: Conceptualization, Data curation, Investigation, Methodology, Software, Writing – review & editing, Resources. LC: Conceptualization, Data curation, Investigation, Methodology, Writing – review & editing, Resources. JC: Methodology, Writing – review & editing, Investigation, Resources, Conceptualization, Data curation. NH: Conceptualization, Data curation, Investigation, Methodology, Resources, Validation, Writing – review & editing, Software, Supervision. LI: Conceptualization, Investigation, Methodology, Writing – review & editing, Resources, Supervision. AJ: Conceptualization, Data curation, Investigation, Methodology, Software, Validation, Writing – review & editing, Resources. MM: Conceptualization, Data curation, Investigation, Methodology, Software, Validation, Writing – review & editing, Resources. BM: Conceptualization, Data curation, Investigation, Methodology, Software, Validation, Writing – review & editing, Resources. AN: Conceptualization, Data curation, Investigation, Methodology, Software, Validation, Writing – review & editing, Resources. DP: Conceptualization, Investigation, Methodology, Writing – review & editing, Data curation, Resources. AP: Conceptualization, Data curation, Investigation, Methodology, Software, Validation, Writing – review & editing, Resources. GS: Conceptualization, Investigation, Methodology, Supervision, Writing – review & editing, Resources. AS: Conceptualization, Investigation, Supervision, Writing – review & editing, Methodology, Resources. SU: Conceptualization, Data curation, Investigation, Methodology, Software, Validation, Writing – review & editing, Resources. AW: Conceptualization, Data curation, Investigation, Methodology, Software, Validation, Writing – review & editing, Resources. JS-L: Conceptualization, Data curation, Investigation, Methodology, Resources, Software, Supervision, Validation, Writing – review & editing. TW: Conceptualization, Data curation, Investigation, Methodology, Resources, Software, Supervision, Validation, Writing – original draft, Writing – review & editing.
